# Development and Evaluation of Low Phytic Acid Soybean by siRNA Triggered Seed Specific Silencing of *Inositol Polyphosphate 6-/3-/5-Kinase* Gene

**DOI:** 10.3389/fpls.2018.00804

**Published:** 2018-06-14

**Authors:** Mansi Punjabi, Navneeta Bharadvaja, Monica Jolly, Anil Dahuja, Archana Sachdev

**Affiliations:** ^1^Department of Biotechnology, Delhi Technological University, New Delhi, India; ^2^Division of Biochemistry, Indian Agricultural Research Institute, New Delhi, India

**Keywords:** soybean, low phytic acid, inositol polyphosphate 6-/3-/5-kinase, RNAi silencing, seed-specific, *Agrobacterium*-mediated transformation

## Abstract

Soybean is one of the leading oilseed crop in the world and is showing a remarkable surge in its utilization in formulating animal feeds and supplements. Its dietary consumption, however, is incongruent with its existing industrial demand due to the presence of anti-nutritional factors in sufficiently large amounts. Phytic acid in particular raises concern as it causes a concomitant loss of indigestible complexed minerals and charged proteins in the waste and results in reduced mineral bioavailability in both livestock and humans. Reducing the seed phytate level thus seems indispensable to overcome the nutritional menace associated with soy grain consumption. In order to conceive our objective we designed and expressed a inositol polyphosphate 6-/3-/5-kinase gene-specific RNAi construct in the seeds of Pusa-16 soybean cultivar. We subsequently conducted a genotypic, phenotypic and biochemical analysis of the developed putative transgenic populations and found very low phytic acid levels, moderate accumulation of inorganic phosphate and elevated mineral content in some lines. These low phytic acid lines did not show any reduction in seedling emergence and displayed an overall good agronomic performance.

## Introduction

For the last 50 years, world population multiplied more rapidly than ever before, and is expected to grow over a third by 2050. Therefore, one of the major challenges agriculture will face in the coming decades is to meet the food demand of the growing population. Scientific community and farmers worldwide are seeking an inexpensive source of energy dense and nutrient rich alternative to serve the purpose. Soybean crop due to its unique nutrient profile provide as a promising option to ensure food security in future. United States Department of Agriculture (USDA) estimated that the global soybean production 2017–2018 will be 348.04 million metric tons. Despite such huge production, the presence of natural anti-nutrients, such as inositol hexakisphosphate (Phytic acid, PA), protease inhibitors, lectins, saponins amongst few others, has limited its consumption (Jiang et al., 2013). PA particularly summon attention as it accounts for over 75% of the total seed phosphorous (Raboy et al., 1984) as well as chelate many divalent cations to form insoluble salts at the biological pH (Halsted et al., 1974; Jacobsen and Slotfeldt-Ellingsen, 1983). Its consumption, however, is inevitable as it is associated with protein bodies found in the cotyledon of the soybean seeds (Lott, 1984; Lott et al., 1995; Wada and Lott, 1997). Therefore, feeding on high-phytate soy-based diets are often feared to exacerbate mineral and protein malnutrition. In addition, phytate excreted by livestock grazing on soy-rich forage contribute significantly to the environmental phosphorus load (Brinch-Pedersen et al., 2000). These problems have provided us with a strong impetus to develop low phytic acid (*lpa*) soybean grains which could contribute in its biofortification.

The first generation of *lpa* crops were developed using classical breeding. With advancement in technology these efforts were augmented by the aid of forward genetics. Mutations that block the synthesis or accumulation of PA during seed development have been isolated in the past in a variety of crop species (Maize, Raboy et al., 2000; Shi et al., 2005; Wheat, Guttieri et al., 2004; Barley, Larson et al., 1998; Rasmussen and Hatzack, 1998; and Rice, Larson et al., 2000; Liu et al., 2007). *Lpa* mutant lines CX1834 (Wilcox et al., 2000) and LR33 (Hitz et al., 2002) were developed in soybean and made available for public breeding efforts. Unfortunately, the crops were unsuccessful due to their poor agronomic performance which called in for a different approach.

Research in the past has evidenced plant genetic engineering techniques to hold unprecedented potential for crop improvement. The first transgenic strategy that was followed to develop *lpa* soybean was the accumulation of microbial phytases in their seeds (Li et al., 1997; Denbow et al., 1998). However, the cost and the labor incurred for its processing prior to consumption made it an economically unviable alternative. Successively, a sustainable approach directing expression of soybean phytase during embryo development at the site of PA synthesis/storage was followed (Hegeman and Grabau, 2001; Chiera et al., 2004). But only a maximum reduction of 25% in PA level was attained by this method which is significantly less than what has been achieved previously. Therefore, to further reduce the PA level albeit optimally, we thought of genetically engineering soybean by exploring RNA interference (RNAi) technique of reverse genetics, which has recently been used to generate *lpa* rice (Ali et al., 2013a,b) and wheat (Bhati et al., 2016). Nunes et al., 2006 achieved upto 95% reduction in soybean seed PA by RNAi but at the cost of embryo abortion in the progeny zygotes. The observed effect can partly be accounted for by firstly, the choice of gene and secondly, the promoter used for expression of the transgene. Nunes in order to manipulate PA biosynthesis, followed inhibition of the first step which is suggested to be the most effective strategy. However, suppressing *myo*-inositol-1-phosphate synthase (*GmMIPS*) expression may lead to critical alterations in seed PA biosynthesis (Kuwano et al., 2009), subsequently disturbing the cellular phosphorus and inositol homeostasis. Therefore, in our study we decided to chose one of the late PA pathway enzyme, inositol polyphosphate 6-/3-/5-kinase (*GmIPK2*) as the target gene. Due to multiple specificity of this enzyme, participating in sequential phosphorylation of 1D-myo-inositol-1,4,5-trisphosphate to 1D-myo-inositol-1,3,4,5,6-penta*kis*-phosphate and its strategic position in the PA biosynthesis pathway we hypothesize that it shall be successful in achieving a greater but optimal level of PA reduction. Further, Nunes et al. (2006) used a constitutive promoter CaMV35S which resulted in a strong expression in vegetative tissues other than developing seeds. From this we conclude that the promoter we use for expression should be active only in developing seeds, the storage site of PA. Also, we kept in mind that the promoter we use should have the same temporal and spatial activity in the seed as *GmIPK2* to achieve a critical level of suppression. With regard to this purpose, we selected the promoter of reserve protein vicilin, located in protein bodies which is the same site as *GmIPK2* gene expression to drive our RNAi construct.

So far there is no report on successful recovery of fertile *lpa* transgenics of Indian soybean cultivars. In this study, we were able to generate *lpa* lines of *Glycine max* Pusa 16 that along with reduction in phytate levels displayed an increase in available phosphorous and few other important mineral elements assayed. In addition, these *lpa* transgenics that we developed can provide a valuable system to study seed PA synthesis and can aid in developing markers for the *lpa* trait in future.

## Materials and Methods

### Plant Material

*Glycine max* [L.] Merr. cv. Pusa-16 procured from Division of Genetics, IARI, New Delhi, India was used for isolation of *GmIPK2* gene and as the recipient genotype for subsequent genetic modification. Quantitative characteristics of this cultivar grown primarily in northern plain and hill zones of India have been described in studies made by Karnwal and Singh (2009) and Ramteke et al. (2010; Ramteke and Murlidharan, 2012).

### Construction of RNAi Expression Vector

**FIGURE 1:**
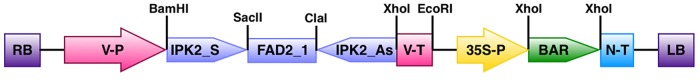
Map showing the T-DNA region of binary vector pCWAK-ipk2. RB: right border; V-P: vicilin promoter; IPK2_S: *IPK2* sense fragment; FAD2_1: fatty acid oley1 Δ12 desaturase gene intron; IPK2_AS: *IPK2* antisense fragment; V-T: vicilin terminator; 35S-P: cauliflower mosaic virus 35S promoter; BAR: bialaphos resistance gene; N-T: nopaline synthase terminator; LB: left border.

Based on the transcript sequence of soybean *IPK2* gene available in the plant comparative genomics portal Phytozome v9.1 (Glyma.12G240900) we designed primers to amplify and clone a 305 bp fragment including 250 bp of its 3′ end coding sequence and a conserved sequence of 55 bp from its 3′ untranslated region. This nucleotide fragment was used to amplify and directionally clone sense (*GmIPK2_S;* FP 5′-CGCGGATCCGCGTTGCAGAAGCTCAAG-3′ and RP 5′-TCCCCGCGGGG AGCGACACTAATTCAAG-3′) and anti-sense (*GmIPK2_As;* FP 5′-CCGCTCGAGCGGAACGTC TTCGAGTTC-3′ and RP 5′-CCATCGATGGCGCTGTGATTAAGTTCGTA-3′) strands around a 395 bp spacer of soybean fatty acid oley1Δ12 desaturase gene intron (*GmFad2-1*; FP 5′-TCCCCG CGGGGAAGGTCTGTCTTATTTTGAATC-3′ and RP 5′-CCATCGATGGTATACCGCACTAGT AAACCAC-3′) cloned in pCR2.1-TOPO cloning vector to form a hairpin (ihp) structure which was confirmed by automated sequencing. The binary vector pCWAK that will carry this silencing cassette was constructed inhouse in a previous work by ligating fragments from the expression vector pCW66 and binary vector pAKVS both kindly obtained from Dr. Craig Atkins Laboratory, University of Western Australia, Australia. The final RNAi construct pCWAK-ipk2 was generated by restriction cloning (*BamHI/XhoI*) of ihp cassette in pCWAK under the control of soybean seed specific *vicilin* gene promoter and terminator (**Figure 1**). The plasmid pCWAK-ipk2 also bears phosphinothricin acetyltransferase (PAT) encoding marker gene *bar* within the T-borders driven by the cauliflower mosaic virus (CaMV) 35S promoter to confer tolerance to the herbicide glufosinate for transgenic selection. After extensive verification of pCWAK-ipk2 by restriction digestion, we subsequently mobilized the binary construct into disarmed *Agrobacterium tumefaciens* strain EHA105 by triparental mating using *Escherichia coli DH5α* (pRK2013) helper strain for further use in preliminary transformation experiments.

### Generation of Transgenic Plants Expressing RNAi Construct

**FIGURE 2:**
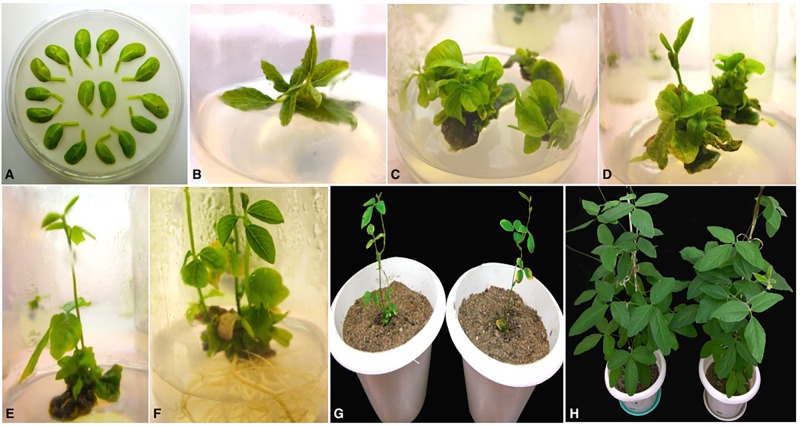
Different stages of *Agrobacterium*-mediated cotyledonary-node transformation of soybean cv. Pusa 16. **(A)** Co-cultured wounded cotyledonary-node explants **(B)** shoot induction from axillary meristem on SIM after 2 weeks; **(C)** shoot development with 4 mg/l glufosinate selection on SIM after 4 weeks of culture; **(D)** shoot elongation with 5 mg/l glufosinate selection on SEM after 6 weeks of culture; **(E)** elongated, glufosinate-resistant shoot ready for transfer to RM after 9 weeks of culture; **(F)** root development on RM; **(G)** acclimatization of plantlets in sterilized pot mix; **(H)** mature T_0_ transgenic plants.

Soybean transformation was performed following *Agrobacterium*-mediated cotyledonary-node method described by Zhang et al., 1999 and Paz et al., 2004 with minor modifications. Briefly, sterilized soybean seeds germinated on half B5 medium were excised to derive two cotyledonary explants per seed and infected with *A. tumefaciens* harboring the RNAi construct (**Figure 2A**). Following 3 days of co-cultivation in dark in the presence of thiol compounds (3.3 mM L-cysteine, 1 mM dithiothreitol, 1 mM sodium thiosulfate) to improve cell transformation efficiency (Olhoft and Somers, 2001; Olhoft et al., 2001), the explants were sub-cultured twice for 12–14 days on full B5 medium (shoot induction medium, SIM) supplemented with 4 mg/l glufosinate for selective shoot induction (**Figures 2B,C**). Four weeks later, healthy explants were transferred to full MS medium (shoot elongation medium, SEM) containing 5 mg/l glufosinate for continued selection and sub-cultured in fresh medium every 2 weeks until shootlets approximately 2–3 cm high were developed (**Figures 2D,E**). Elongated shootlets were subsequently induced to develop roots by the application of 2mg/l Indole-3-butyric acid (IBA; auxin phytohormone) supplemented in half MS medium (rooting medium, RM) (**Figure 2F**). Well rooted plantlets were transplanted to soil pots for hardening and grown to maturity under 16 h/8 h light/dark regime at National Phytotron Facility, IARI, Delhi, India (**Figures 2G,H**).

### Transgene Integration Analysis

#### PCR Examination

The putative transformants (T_0_) that survived hardening were selected for primary screening of transgene integration through PCR analysis. Total genomic DNA was isolated from their leaves and the leaves of non-transformed control plants using genomic DNA mini kit (plant) following manufacturer’s (Geneaid, Taiwan) protocol. *GmIPK2_S* forward (FP 5′-CGCGGATCCGCGTTGC AGAAGCTCAAG-3′) and *GmFAD2-1 reverse* (RP 5′-CCATCGATGGTATACCGCACTAGTAA ACCAC-3′) primers were used to amplify a 700 bp fragment using thermal cycling conditions of 1 min at 94°C; 30 cycles of 30 s at 94°C, 30 s at 62°C, 30 s at 72°C; and a final extension of 10 min at 72°C. The amplicon generated was purified and sequenced for confirmation.

#### Segregation Analysis

Segregation analysis of the transgene was performed by studying *bar* gene expression in the T_1_ progenies of each independent transformation events characterized. Seeds from these events were germinated in pots filled with Vermiculite, Cocopeat and Sand mixture (1:1:1). Leaf tissues were collected from 30 days old seedlings for genomic DNA isolation and PCR amplification was carried out using *bar* specific primers (FP 5′-GAACGACGCCCGGCCGACAT-3′ and RP 5′-GTCCAGC TGCCAGAAACCCAC-3′) under thermal cycling conditions of 1 min at 94°C; 30 cycles of 30 s at 94°C, 30 s at 65°C, 1 min at 72°C; and a final extension of 3 min at 72°C, to amplify a 500 bp fragment.

#### Analysis by Southern Blotting

Southern hybridization analysis was carried out on plants cultivated from seeds of PCR positive T_0_ lines to confirm stable T-DNA integration and to estimate the number of copies inserted. Twenty micrograms of genomic DNA from each sample was digested with *PstI* which does not cut the T-DNA region. *XhoI* digested pCWAK-IPK2 plasmid DNA was used as the positive control. The restricted samples were separated by electrophoresis on 0.8% agarose gel and transferred onto positively charged nylon membrane (Axiva, India) by capillary blotting following the method described by Sambrook et al. (1989). The membrane was UV cross-linked and subsequently hybridized with *bar*-specific probe at 42°C for 16 h, biotin labeled using Biotin DecaLabel DNA Labeling Kit (Thermo Scientific, United States). The hybridized membrane was washed and detected using Biotin Chromogenic Detection Kit according to the protocol described by the manufacturers (Thermo Scientific, United States).

### Transcript Analysis by Quantitative Real-Time PCR

Southern positive T_2_ plants were further analyzed by semiquantitative RT-PCR and quantitative real-time PCR (qRT-PCR) to estimate *IPK2* transcript levels. Total RNA was isolated from fresh soybean seed (8–10 mm) tissue of T_2_ transgenic and non-transgenic plants using TRI Reagent (Sigma-Aldrich, United States). First-strand cDNA was synthesized from 5 μg of DNaseI-treated total RNA using M-MLV Reverse Transcriptase (Promega, United States) following manufacturer’s instructions. qRT-PCR analysis was subsequently performed following SYBR Green (DyNAmo Flash SYBER Green qPCR Kit, Thermo Scientific, United States) chemistry on PikoReal 96 Real-Time PCR platform (Thermo Scientific, United States). Gene-specific primers were designed to amplify a 124 bp fragment from its conserved region (*qIPK1*F 5′-GGAGCGCTTGCAGAAGC-3′, *qIPK1*R5′-GACCAGAGGGTTGGT AGC-3′). To normalize the variance among samples, house-keeping gene phosphoenolpyruvate carboxylase (*PEPCo*) (q*PEPCo*F5′-CATGCACCAAAGGGTGTTTT-3′, q*PEPCo*R 5′-TTTTGCG GCAGCTATCTCTC-3′) was used as endogenous control. Amplification was achieved by a two-step PCR reaction with an initial denaturation at 94°C for 4 min followed by 40 cycles of 94°C for 30 s and annealing/extension at 60°C for 30 s. Triplicate quantitative assays were performed on biological triplate corresponding to each sample. Relative mRNA abundance was calculated using the 2^-ΔΔ*CT*^ method described by Livak and Schmittgen, 2001. The specificity of each unique amplification product was determined by melting curve analysis.

### Estimation of Seed Phosphorus Levels

Total phosphorous in the seeds was determined colorimetrically by a method described by Chen et al. (1956). Samples (transgenic and non-transgenic control) for the assay were prepared by wet ashing dried ground seeds (250 mg) in 2 ml of concentrated sulfuric acid (Larson et al., 2000; Raboy et al., 2000).

For the analysis of inorganic phosphorus (Pi) levels, we followed a protocol described by Al-Amery et al. (2015) which uses single seed chips (1–2 mg) derived from cotyledonary segment opposite to the embryonic axis. Briefly, seed samples were extracted using 50 μl buffer (25 mM magnesium chloride and 12.5% trichloroacetic acid) for 14–16 h at 37°C with gentle shaking. 10 μl subsample of extracts were loaded in triplicates onto a 96 well plate and diluted with 90 μl water. Each sample was incubated with 100 μl of Chen’s reagent at 37°C for 1 h and the absorbance was read at 882 nm on GloMax^®^-Multi Detection System (Promega, United States).

### Analysis of Phytic Acid Concentration by HPLC

Phytic acid estimation in transgenic seeds was performed by reversed-phase high-performance liquid chromatography (RP-HPLC) as described by Lehrfeld (1989) with some modifications (Pandey et al., 2016). 500 mg dried seed tissue was homogenized in a pestle-mortar, hexane-defatted and subsequently extracted with 0.78 M HCl by sonication (3 min) and mechanical agitation for 1 h at 250 rpm. The extract was centrifuged and one part of clear supernatant was mixed with four parts of HPLC grade water. The diluted sample was passed through conditioned SAX column (Hypersep, Thermo Scientific, United States) connected to a vacuum manifold (Millipore, United States) and eluted with 2 M HCl. The filtrate was evaporated in a vacuum rotary evaporator (Hei-VAP Value Digital G3, Heidolph, Germany), re-dissolved in 1 ml of mobile phase (acetonitrile, formic acid and tetrabutylammonium hydroxide, 4.8:5.1:0.1, v/v/v) and filtered using a 0.2 micron PVDF syringe filter (Millipore, United States). For HPLC analysis, sample was injected onto the C18 RP-column (250 × 4.6 mm, 5 μ; Shimadzu, Japan) equilibrated with isocratic mobile phase at a flow rate of 1.0 ml min^-1^. PA signals were monitored with a UV-VIS photodiode array detector (SPDM-20Avp, Shimadzu, Japan) at a wavelength of 197 nm. The PA concentration was calculated using the calibration curve prepared with a PA dodecasodium salt standard (Sigma-Aldrich). The software used for analysis was LC-Solutions (version 1.25).

### *In Vitro* Bioavailability Assay

Mature transgenic and non-transgenic seeds were milled (Wiley, Thomas Scientific, United States), weighed (1 g) and digested in triplicates under simulated gastrointestinal conditions following method described by Kiers et al., 2000 to determine the bioavailability of iron (Fe), zinc (Zn), and calcium (Ca). The digest was centrifuged at 9000 rpm for 15 min and supernatant obtained was passed through a 0.45 mm filter. The filtrate was analyzed for Fe, Zn, and Ca using an Atomic Absorption Spectrophotometer (AAnalyst 200, Perkin Elmer, United States). A blank consisting of distilled water was processed in a similar manner and used for sample correction.

### Agronomic Evaluation of Transgenic Plants

#### Germination Assay for Seed Viability

The germination capacity of T_3_ transgenic seeds as compared to non-transgenic control was assessed by performing controlled germination test (CGT) described by Campion et al., 2009. The seeds were immersed under water for 8 h at 28°C and then transferred to fresh water for an additional 12 h. Following incubation, seeds were rinsed three to four times in distilled water and subsequently germinated in petri dishes lined with filter paper soaked in distilled water under a photoperiod of 28°C 8-h dark and 30°C 16-h light. We also monitored seed vigor by using the standard accelerated aging test (AAT) test. In this we subjected unimbibed seeds to conditions of high temperature (42°C) and high relative humidity (90%) for 72 h and then removed them from the stress conditions and placed under optimum germination conditions.

#### Phenotypic Analysis

Different morphological traits of transgenic plants were evaluated under controlled conditions to capitulate the influence of transgene integration on phenotype. Mature transgenic plants were harvested and evaluated with respect to the non-transgenic control for plant height, number of pods, number of seeds, seed dry weight, stem length and root length. The height of individual plant was measured as the distance from the soil surface to the tip of the plant. On maturity, upto 100 seeds were harvested and their mean dry weight (SDW) was recorded after air-drying them in oven at 60°C for 72–96 h. Five randomly chosen plants from each transgenic line were evaluated for each parameter studied.

### Statistical Analysis

Every experiment was carried out in biological and experimental replications (three to six) for each non-transgenic control and transgenic sample and represented as mean ± standard error. All statistical evaluations were performed using SAS software (version 9.2). Segregation patterns analyzed with chi-square (χ^2^) goodness of fit were tested at 5% level of significance. We also conducted ANOVA for agronomic traits of transgenics and non-transgenic control and compared the group means by Tukey’s test at 5% level of significance.

## Results

### Transgene Integration and Segregation Analysis

**FIGURE 3:**
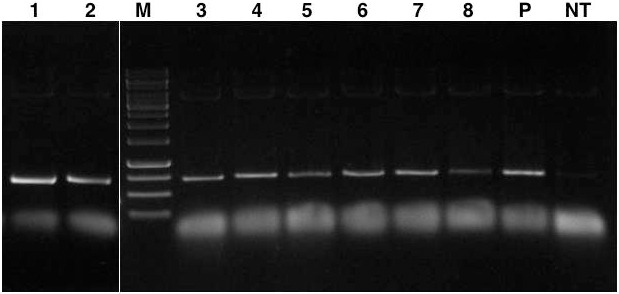
PCR amplification of ∼700 bp *GmIPK2_S* gene plus *GmFAD2-1* intron fragment from genomic DNA of T_0_ transgenic plants. Lanes, M: 1 kb DNA ladder; 1–8: genomic DNA from each transformation event characterized; P: pCWAK-ipk2 plasmid DNA (positive control); NT: genomic DNA from non-transformed plant (negative control). Representative lanes were spliced from the original gel (Supplementary Figure S1).

A preliminary screening was conducted on the T_0_ plants to identify putative *lpa* transgenic lines by PCR amplification of ∼700 bp fragment of ihp construct. The assay characterized eight independent transformation events each showing the expected amplicon size which was absent in the case of untransformed control plants (**Figure 3**).

Seeds from each independent event identified were grown successfully under containment conditions to obtain T_1_ progeny plants by self pollination. All the derived progenies appeared phenotypically normal and fertile. We performed segregation analysis on them by studying *bar* gene expression and found that five of the events (P2, P4, P5, P6, and P8) held a good fit to the stable Mendelian inheritance of a single locus (3:1) (**Table 1**) whilst three (P1, P3, and P7) of them showed a segregation ratio of two transgene loci (15:1).

**Table 1:** Segregation of *bar* gene amongst T_1_ progenies of eight independent transformation events characterized in soybean cv. Pusa 16.

Transgenic events	No. of seeds tested (n)	No. of seedlings				Ratio	Chi-square value (χ^2^)^∗^
		PCR +		PCR -			
		Obs.	Exp.	Obs.	Exp.		
P1	55	50	51.56	5	3.43	15:1	0.766
P2	47	37	35.25	10	11.75	3:1	0.347
P3	58	53	54.38	5	3.62	15:1	0.561
P4	44	35	33.00	9	11.00	3:1	0.485
P5	49	39	36.75	10	12.25	3:1	0.551
P6	52	36	39.00	16	13.00	3:1	0.923
P7	56	51	52.50	5	3.5	15:1	0.686
P8	53	37	39.75	16	13.25	3:1	0.773

*^∗^$\chi_{\mathrm{20.05}}^{2}$; df1 = 3.841.*

**FIGURE 4:**
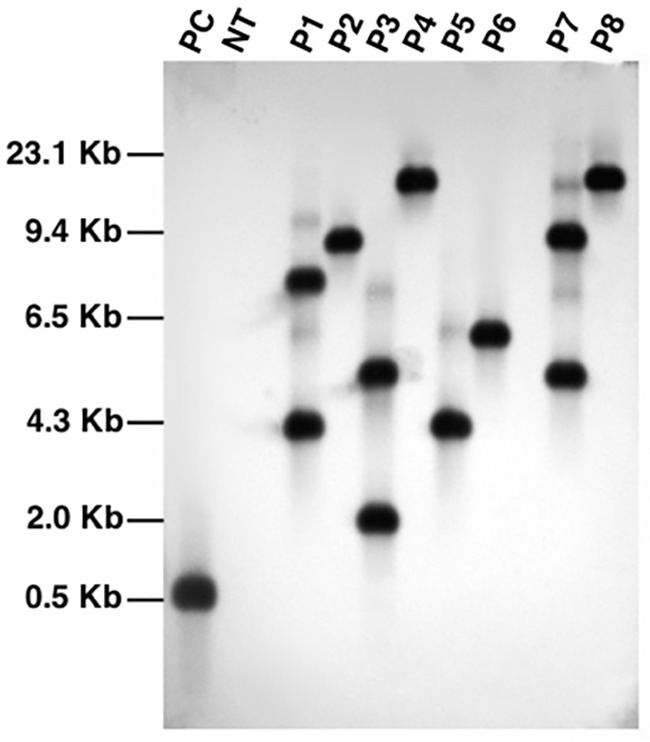
Southern blot analysis of PCR characterized transgenic events using *bar* gene specific probe. Lanes, PC: plasmid pCWAK-ipk2 (positive control); NT: genomic DNA from non-transformed plant (negative control); P1-P8: genomic DNA of independent transgenic events showing hybridization signals indicating transgene integration.

We further confirmed integration of the transgene cassette by southern blot analysis using *bar* gene probe (**Figure 4**). The genomic DNA was digested with *PstI* endonuclease which is not present within the T-DNA of the construct. Upon detection, all the transgenic lines analyzed showed a distinct pattern of seperation which indicate that each plant originated from an independent transformation event. The blot showed that all the fragments were above 3 kb in size which suggest that the transgenic lines carry intact copies of T-DNA because the shortest fragments in each line were longer than the T-DNA region (∼2.8 kb). The blot also confirmed single copy integration of target gene into the genome of transgenic events P2, P4, P5, P6, and P8. Since single copy insertions are always desirable, T_2_ generation plants were cultivated from seeds of these five characterized events and PCR screened to select homozygous lines. A positive amplification in all the tested T_2_ plants derived from the T_1_ parents, P2-45 and P6-39, suggest their homozygous nature and thus seeds collected from only these parents were selected to characterize *lpa* trait by further analysis.

### Expression Analysis of Transgenic Plants

**FIGURE 5:**
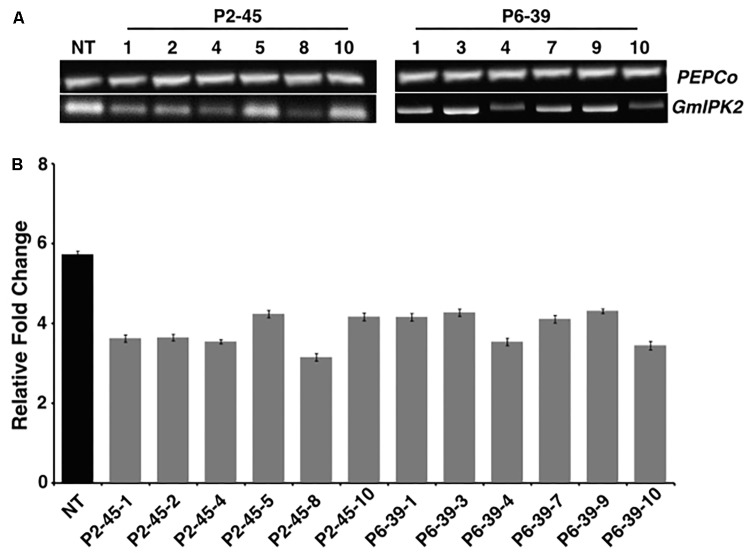
Expression analysis of transgenic soybean seeds. **(A)** RT-PCR amplification showing variation in *GmIPK2* transcripts in T_3_ seeds of P2-45 and P6-39 compared to the *PEPCo* internal control (NT: Non-transformed plant). Representative lanes were spliced from the original gel (Supplementary Figure S2). **(B)** Relative fold change measured by qRT-PCR in the above samples indicating varied levels of silencing with maximum reduction observed in P2-45-8. The data presented is mean of technical triplicates corresponding to each biological replicate (*n* = 3) with error bars indicating standard deviation (SD).

To assess the level of reduction in *GmIPK2* expression in transgenic seeds with respect to non-transgenic control, we carried out RT-PCR and qRT-PCR analysis. From RT-PCR results, we observed a variable reduction in the expression of *GmIPK2* gene with no variation in *PEPCo* transcript expression between the non-transgenic and transgenic plants thus supporting its use as a stable endogenous reference (**Figure 5A**). We further quantified the variations in *GmIPK2* transcript levels by qRT-PCR and found a maximum reduction of 2.79-fold and 2.56-fold in T_3_ developing seeds from the transgenic lines P2-45-8 and P6-39-10, respectively (**Figure 5B**) at *P* ≥ 0.05. We also studied *GmIPK2* expression in other plant tissues of these two lines and found no variation in its transcript levels with respect to the control plants (Supplementary Figures S3, S4) which suggest its successful silencing only in the seeds.

### Seed Phytic Acid Quantification

**FIGURE 6:**
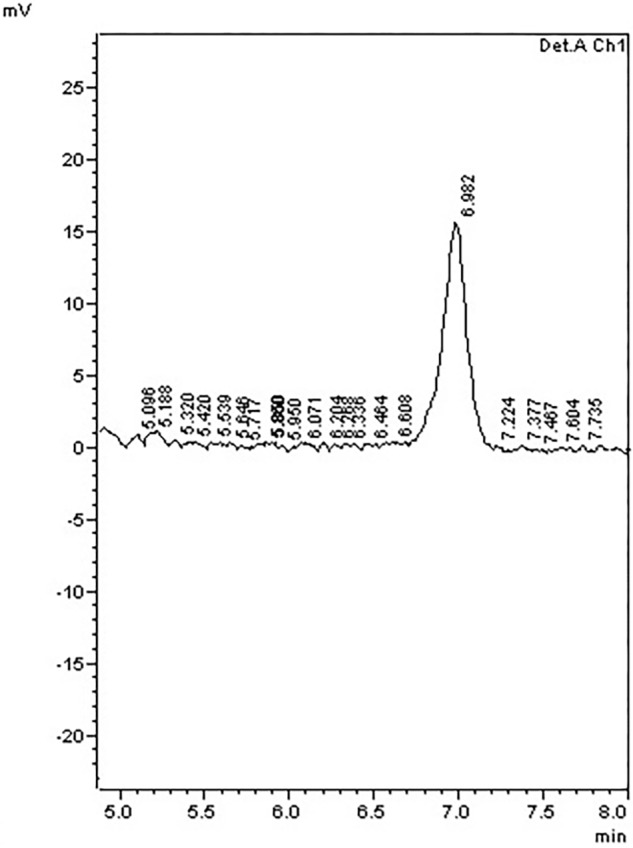
HPLC phytic acid peaks of non-transgenic control seed extracts.

**FIGURE 7:**
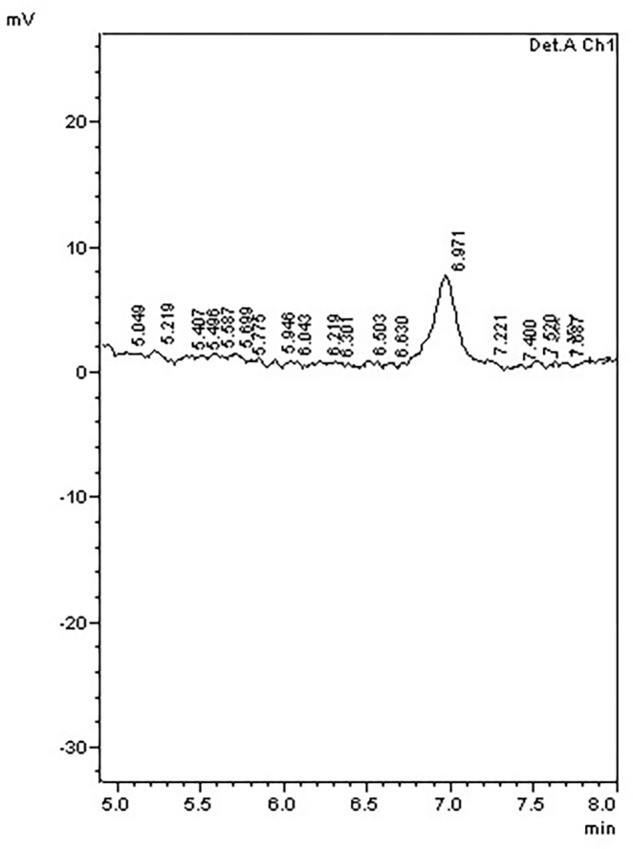
HPLC phytic acid peaks of seed extracts from transgenic line P2-45-8.

**FIGURE 8:**
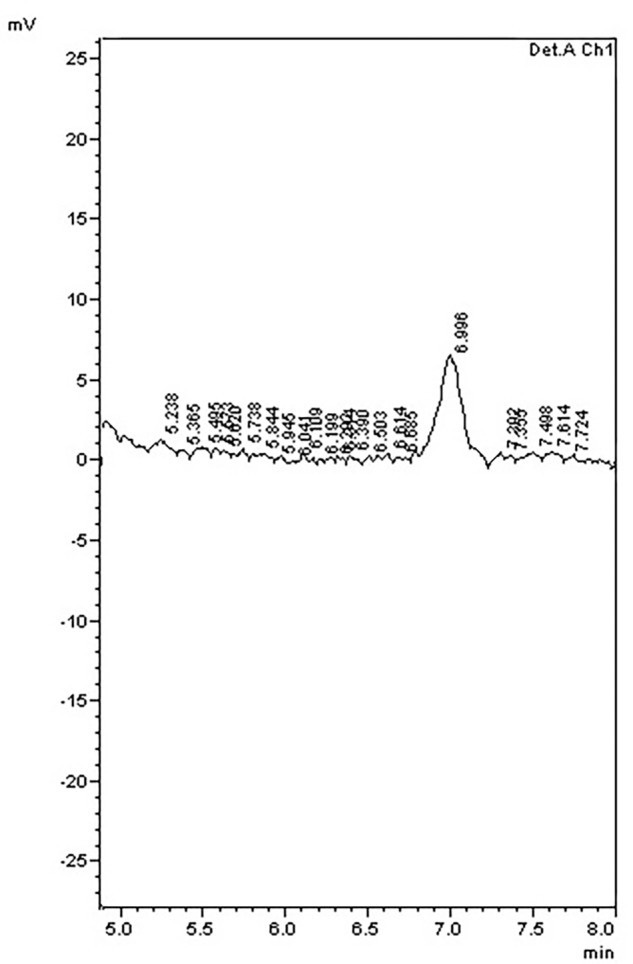
HPLC phytic acid peaks of seed extracts from transgenic line P6-39-10.

**FIGURE 9:**
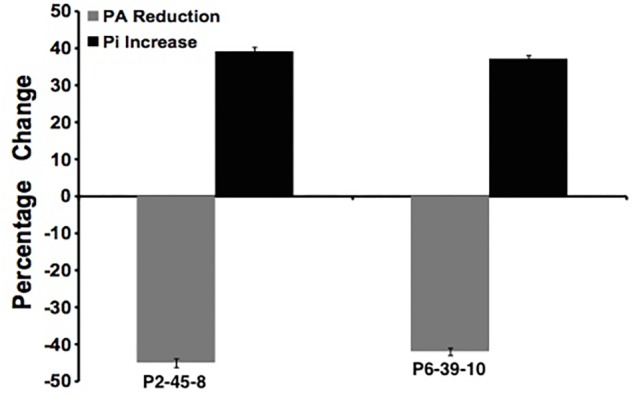
Percentage reduction in phytate (gray column) and increase in free phosphate (black column) in P2-45-8 and P6-39-10 transgenic lines.

To further examine the effect of *GmIPK2* silencing on PA synthesis, we quantified PA content in mature grain extracts of above two lines and non-transgenic samples by HPLC analysis. The chromatogram obtained by scanning the samples at 197 nm displayed PA retention peaks around 7.006 ± 0.09 min, the area under which was used to compute the level of PA. A decrease in the PA content was observed in T_3_ seeds compared to control which displayed larger peak area indicating its higher concentration in the seeds (**Figures 6–8**). The mean PA content was 3.48 g 100g^-1^ for non-transgenic seeds against 1.91 g 100 g^-1^ and 2.02 g 100 g^-1^ for the seeds from transgenic lines P2-45-8 and P6-39-10, showing a maximal reduction of 45 and 42%, respectively (**Figure 9**).

### Analysis of Seed Phosphorus Levels

We estimated the total and free phosphorus (Pi) levels in transgenic as well as non-transgenic seeds to correlate it with the reduction in PA content. No significant difference in total seed phosphorus (P) was observed for the RNAi lines as compared to the control. The average total phosphorus content of T_3_ seeds was 5.82 mg g^-1^ (P2-45-8) and 5.75 mg g^-1^ (P6-39-10), which was observed closely similar to that of non-transgenic seeds, 5.89 mg g^-1^. However, a significant increase of 39 and 37% in the concentration of Pi was recorded in the T_3_ seeds obtained from transgenic lines P2-45-8 and P6-39-10, respectively, which gave a dark blue reaction with chen’s reagent, confirming a reduction in the content of its principal storage form PA (**Figure 9**).

### Quantification of Mineral Content in Seeds

In order to determine whether mitigation of PA improved the mineral bioavailability in soy grains, we made a quantitative estimation of micronutrients in transgenic seeds compared to non-transgenic control by following *in vitro* digestion method. From the observations made by atomic absorption spectrophotometer, we noticed an increase in the *in vitro* Fe, Ca, and Zn availability from 54.8, 44.15 and 58.50% for non-transgenic control seeds to 71.1, 59.2, 66.9% for P2-45-8 and 70.7, 58.42, 64.50% for P6-39-10 seeds (**Table 2**). As mentioned before, PA binding with minerals result in formation of insoluble salts with poor bioavailability, our result suggests that the mineral bioavailability can significantly be improved by reduction of PA in *lpa* transgenic lines.

**Table 2:** *In vitro* bioavailability assay of T_3_ seeds.

Minerals	*In vitro* availability (%)		
	Non-transgenic (control)	P2-45-8	P6-39-10
Iron	54.80 ± 0.92	71.10 ± 1.11	70.70 ± 1.05
Calcium	44.15 ± 1.04	59.20 ± 0.78	58.42 ± 0.98
Zinc	58.50 ± 0.85	66.90 ± 0.93	64.50 ± 1.07

*Data represent means ± SD, n = 3.*

### Seed Germination and Morphological Trait Analysis of Transgenic Plants

Decrease in seed PA concentration can be accompanied by adverse agronomic consequences. Therefore, we conducted germination tests on the *lpa* seeds collected from our transgenic lines. The CGT test so conducted confirmed that 98% of the seeds possessed good germination potential whereas AAT test established their high vigor (Supplementary Figure S5A). Also, the morphological analysis of seed germination in each of the tests revealed similar phenotypes in both transgenic and control seeds (Supplementary Figure S5B). Besides, since PA also regulates the growth and development of seedlings, we carried out phenotyping on 75 days old plants. The growth of transgenic plants was compared with the growth of control soybean plants under field growth conditions. Interestingly, P2 and P6 plants exhibited an increase in leaf size which could possibly be explained due to increase in available seed P content (*P* < 0.05) (Tubana and George, 1997). No significant differences were observed between wild type and transgenic plants in other agronomic traits investigated (*P* > 0.05) (**Table 3**). This data confirms that *GmIPK2* silenced, *lpa* soybean seeds are capable of generating plants showing normal morphologies and agronomic traits.

**Table 3:** Morphological and yield contributing characters of T_2_ transgenic plants raised in green-house.

Parameters	Non-transgenic (control)	P2-45-8	P6-39-10
Shoot length (cm)	73.40 ± 1.68	72.11 ± 1.42	70.20 ± 1.75
Root length (cm)	30.07 ± 0.15	28.43 ± 0.21	27.64 ± 0.15
Leaf length (cm)	11.20 ± 0.29	12.86 ± 0.18	12.67 ± 0.24
Leaf width (cm)	5.36 ± 0.18	6.01 ± 0.14	5.98 ± 0.17
No. of trifoliates per plant	23.07 ± 0.84	24.07 ± 0.43	23.55 ± 0.26
No. of pods per plant	49.45 ± 0.94	46.50 ± 1.08	45.18 ± 1.24
No. of seeds per pod	2.63 ± 0.08	2.72 ± 0.14	2.54 ± 0.11
100 seeds dry wt (g)	15.21 ± 0.25	14.95 ± 0.14	14.83 ± 0.32

*Data represent means ± SD, n = 6.*

## Discussion

Several mutational and transgenic strategies have been explored to reduce PA in soybean but with undesirable aspects (Meis et al., 2003; Yuan et al., 2007, 2009; Anderson and Fehr, 2008; Maupin and Rainey, 2011; Maupin et al., 2011). We may attribute these negative effects primarily to the constitutive suppression of PA biosynthesis genes which result in an indirect impact on several metabolic processes and signal transduction pathways. Prattley and Stanley (1982) established that the cotyledon of soybean contains 90% of the phytate of the seed, we therefore, expressed our RNAi construct under the control of a cotyledon-specific vicilin promoter (Higgins et al., 1988; Rosche et al., 2002) with the aim to restrict PA perturbations to the seed. As expected, initial characterization of the transgenic genotypes displayed stable integration and inheritance of the transforming DNA with no significant impact on whole plant performance and yield. Segregation patterns observed in the T_1_ progenies of distinguished lineages demonstrate that both homozygous and heterozygous plants were obtained from self-pollinated seeds of most of the events, suggesting that T_0_ plants were uniformly transformed and were heterozygous for the transformed genotype. Transgenic plants containing single copy or double copies of the transforming gene were also characterized by segregation analysis. Hobbs et al., 1993 through their studies established that single copy transgenic plants are always desirable to avoid the possibility of gene silencing during introgression of the transgene into the parental line(s) of hybrids, or into improved open-pollinated varieties, we therefore confirmed these single copy events by performing southern blot before proceeding any further analysis.

We used qPCR to check *GmIPK2* expression in T_3_ seeds as well as other plant tissues. From our results, we observed a varied level of reduction of *GmIPK2* transcripts in seeds of lines P2-45 and P6-39. These variations can be explained by several factors viz. differences in the site of integration on the chromosomal DNA, possible promoter methylation etc. (Day et al., 2000; Van Leeuwen et al., 2001). On the other hand, no *GmIPK2* reduction was detected in other tissues of transgenic and control plants demonstrating seed-specific regulation. We further confirmed the implications of *GmIPK2* silencing by testing the extracts from T_3_ generation seeds for both PA and Pi. A reduction of 45% in the PA content and a 39% increase in Pi levels was found in transgenic line P2-45-8, suggesting a strict correspondence between seed PA and Pi which is in accordance with the previous reports by Larson et al., 2000. Moreover, despite the variations in PA and Pi levels it was found that the mean total P content in the transgenic seeds was unaffected, which suggest that these altered genotypes influence P partitioning to strike a balance for supporting P-related mechanisms in the seed.

Phytic acid represents a major sink for mineral deposition due to its natural strong chelation ability. Research has previously reported the beneficial effects of cereal *lpa* mutants in improved mineral cations bioavailability (Mendoza et al., 1998; Adams et al., 2002; Hambidge et al., 2004, 2005; Linares et al., 2006; Mazariegos et al., 2006; Veum et al., 2007). We therefore analyzed the mineral concentration of transgenic seeds and found that disruption of *GmIPK2* expression caused the Fe, Ca, and Zn contents of seeds to increase. We must also highlight that amongst the three different minerals analyzed, a maximum increase was observed in the levels of Fe followed by Ca and Zn, respectively, supporting the findings of Persson et al., 2009 who suggest that Fe in the seeds is mainly associated with PA.

Phytate reduction in a plant is often negatively correlated with its agronomic performance (Bregitzer and Raboy, 2006) which is initially observed as a direct affect on its seed germination ability. We therefore conducted germination trials on our *lpa* transgenics and found that they show a good response, with seedling emergence almost identical to the non-transgenic parent type. Our results seem to be in agreement with the recent reports of Shi et al., 2007 and Spear and Fehr, 2007 who suggest that high levels of PA are not an absolute requirement for efficient seed germination or emergence. Lastly, we studied the morphological traits of each transgenic line and found that the lines generated from events P1 and P4 produced seeds with reduced size and weight. The lower seed yield may be attributed to reduction in starch accumulation resulting from indirect affect of disturbed Pi homeostasis on a key regulatory enzyme in starch biosynthesis pathway (Plaxton and Preiss, 1987).

From our research, we can assert that *GmIPK2* gene is an appropriate candidate for targeted phytate reduction without any pleotropic effects. Despite our results, there is a need to conduct additional field trials under a variety of conditions, across generations and perform their in depth phenotypic and genotypic analysis in order to conclude the effect of *lpa* trait. Further studies are therefore underway in our laboratory in an effort to establish the same.

## Author Contributions

MP contributed in conception and designing of the study, performed the experiments, acquired the data, performed its analysis and interpretation, and drafted the final manuscript. NB contributed in designing the study, revising the manuscript critically for important intellectual content, and gave final approval of the version to be published. MJ contributed in data acquisition, revising the manuscript critically for important intellectual content, and gave final approval of the version to be published. AD contributed in interpretation of data, revising the manuscript critically for important intellectual content, and gave final approval of the version to be published. AS contributed in conception and designing of the study, revising the manuscript critically for important intellectual content, and gave final approval of the version to be published.

## Conflict of Interest Statement

The authors declare that the research was conducted in the absence of any commercial or financial relationships that could be construed as a potential conflict of interest.
