# Supplementary material for: Development and Evaluation of Low Phytic Acid Soybean by siRNA Triggered Seed Specific Silencing of Inositol Polyphosphate 6-/3-/5-Kinase Gene
Source: Front Plant Sci. 2018 Jun 14;9:804. doi: 10.3389/fpls.2018.00804 (PMC6011814; doi:10.3389/fpls.2018.00804)
Supplement: Supplementary file 1 [file Presentation_1.pdf]

Supplementary Material

Original Research

Mansi Punjabi[1,2], Navneeta Bharadvaja[1], Monica Jolly[2], Anil Dahuja[2], Archana Sachdev*[2]

*** Correspondence:** Archana Sachdev: arcs_bio@yahoo.com

Supplementary Figures

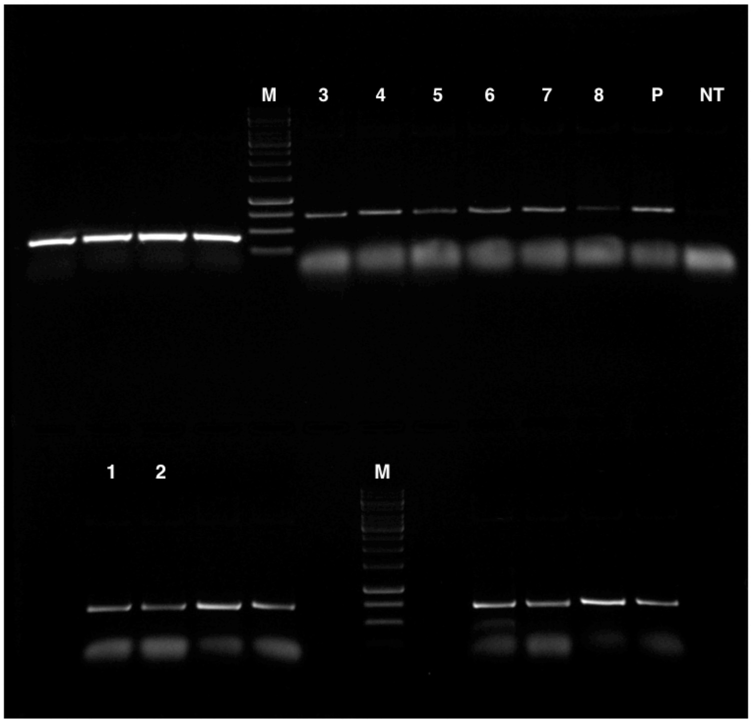

Supplementary Figure S1. PCR amplification of ~700 bp *GmIPK2_S* gene plus *GmFAD2-1* intron fragment from genomic DNA of T_0 transgenic plants. Lanes, M: 1 kb DNA ladder; 1-8: genomic DNA from each transformation event characterized; P: pCWAK-ipk2 plasmid DNA (positive control); NT: genomic DNA from non-transformed plant (negative control).

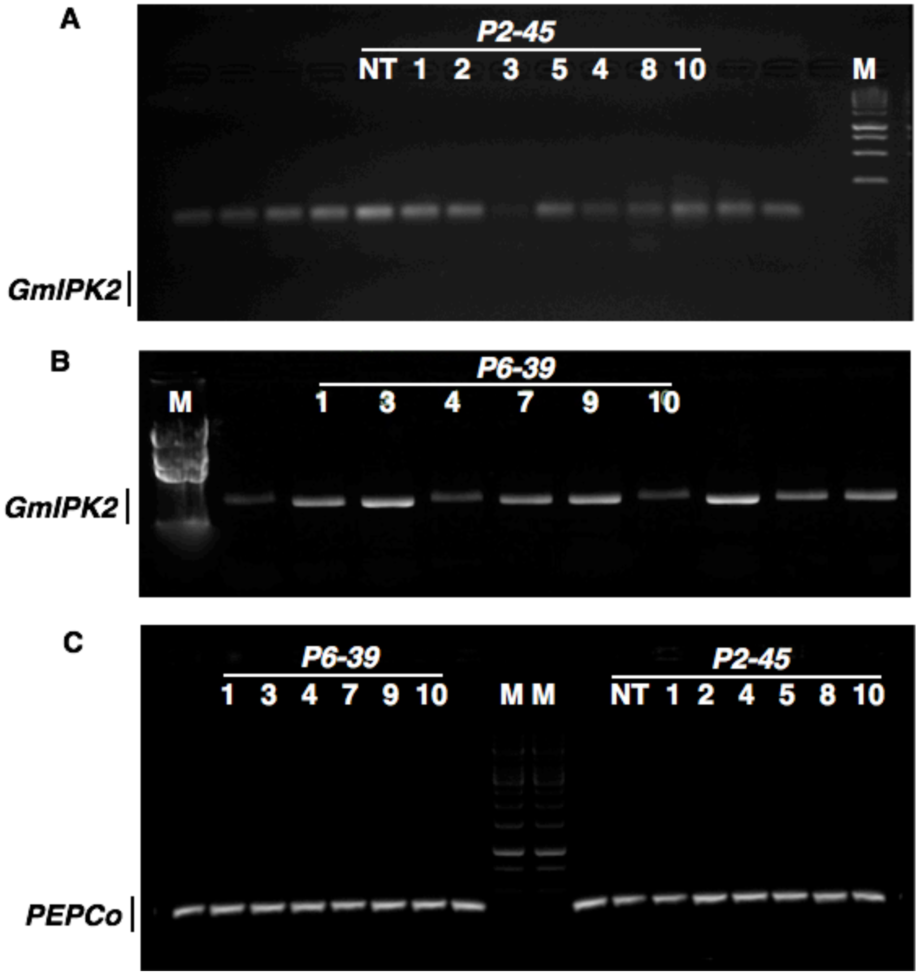

Supplementary Figure S2. RT-PCR amplification showing variation in *GmIPK2* transcripts in T_3 seeds of transgenic events (A) P2-45 and (B) P6-39 compared to (C) the *PEPCo* internal control (NT: Non-transformed plant; M: 1 Kb DNA ladder).

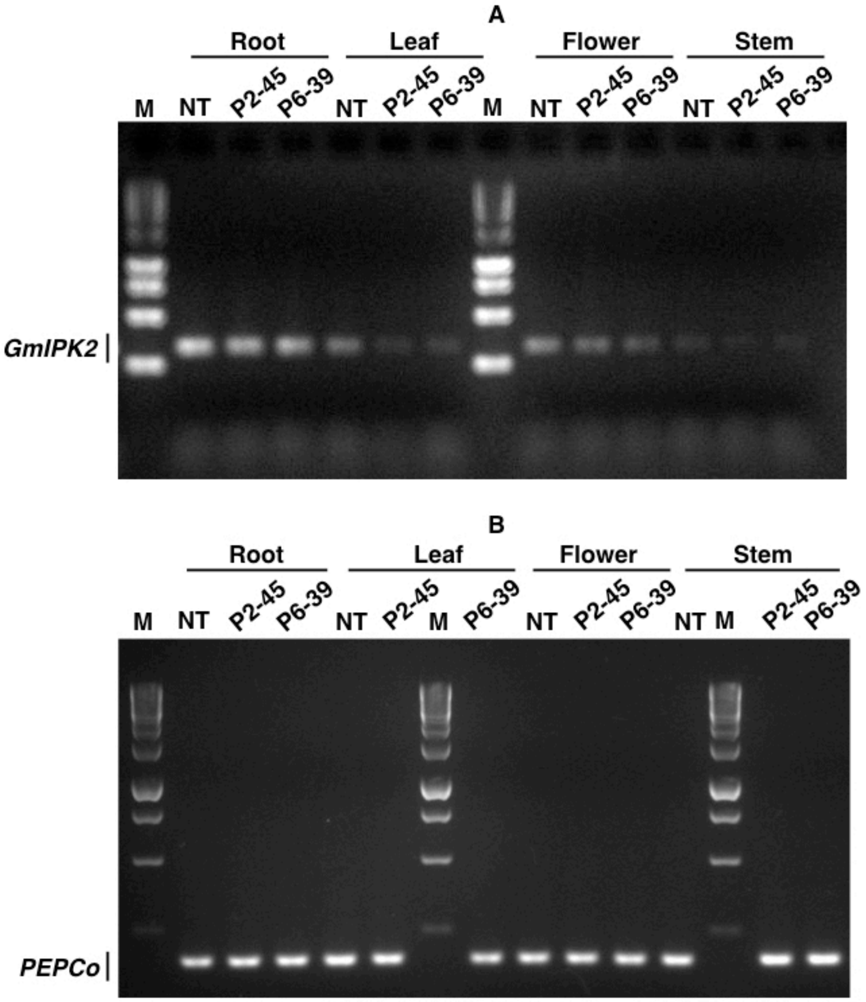

Supplementary Figure S3. RT-PCR amplification showing variation in (A) *GmIPK2* transcripts in different tissues of transgenic events P2-45 and P6-39 compared to (B) the *PEPCo* internal control (NT: Non-transformed plant; M: 1 Kb DNA ladder).

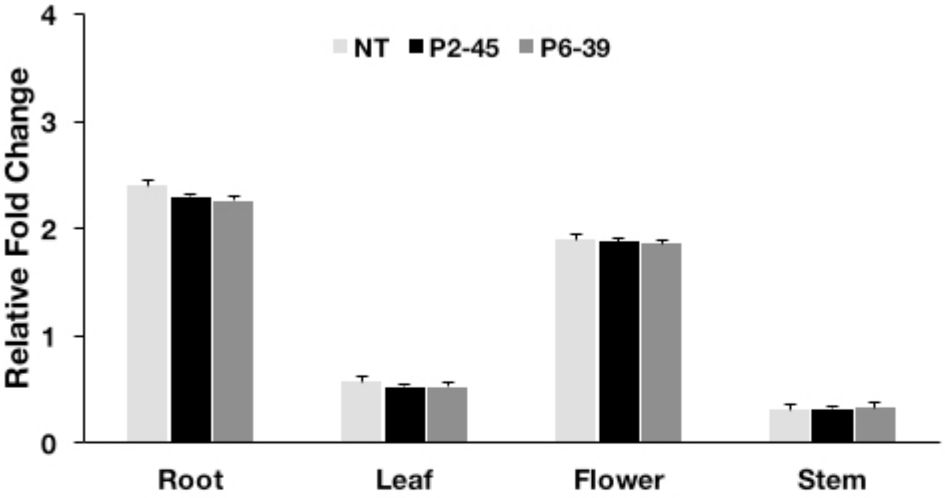

Supplementary Figure S4. Relative fold change measured by qRT-PCR in different tissues of transgenic events P2-45 and P6-39 compared to non-transformed control plants (NT), indicate no variation in the *GmIPK2* transcript level. Each sample was normalized to housekeeping gene *PEPCo*. The data presented is mean of technical triplicates corresponding to each biological replicate (n=3) with error bars indicating standard deviation (SD).

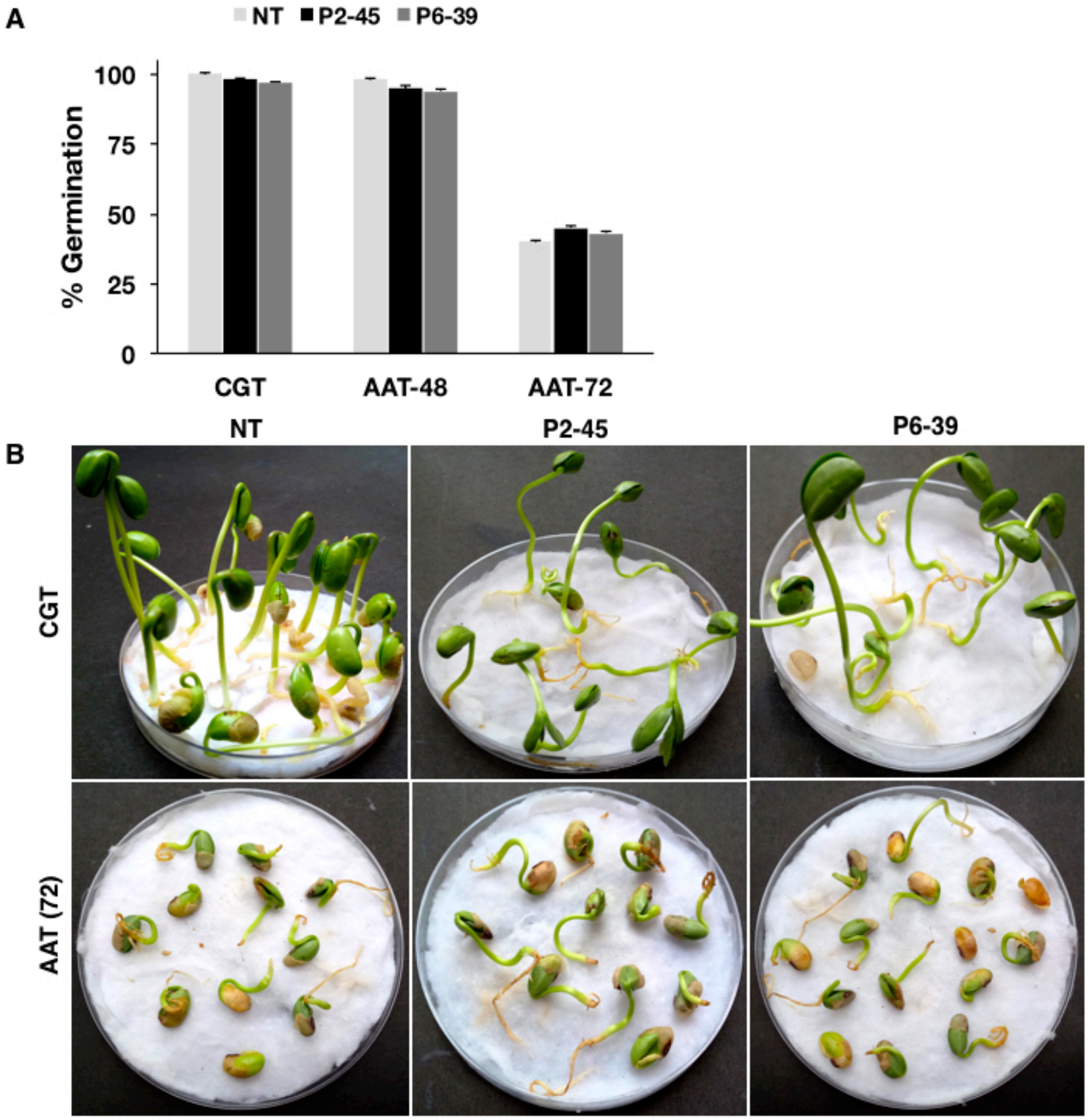

Supplementary Figure S5. Analysis of germination potential in T_3 transgenic lpa seeds compared to non-transgenic seeds. (A) Germination percentage as observed during control germination test (CGT) and accelerated ageing test (AAT) at 48 hrs and 72 hrs time interval in both transgenic (P2-45 and P6-39) and non-transgenic seeds (NT). (B) Morphology of transgenic seeds compared to the non-transgenic control on 5th day of germination during both CGT and AAT.
